# Promising Occupational Safety, Health, and Well-Being Approaches to Explore the Future of Work in the USA: An Editorial

**DOI:** 10.3390/ijerph19031745

**Published:** 2022-02-03

**Authors:** Sara L. Tamers, Jessica M. K. Streit, Casey Chosewood

**Affiliations:** 1 Centers for Disease Control and Prevention, National Institute for Occupational Safety and Health, 395 E Street SW, Washington, DC 20201, USA; 2 Centers for Disease Control and Prevention, National Institute for Occupational Safety and Health, 1090 Tusculum Avenue, Cincinnati, OH 45226, USA; jstreit@cdc.gov; 3 Centers for Disease Control and Prevention, National Institute for Occupational Safety and Health, 1600 Clifton Rd., Atlanta, GA 30329, USA; lchosewood@cdc.gov

## 1. Introduction

The future of work continues to undergo profound and fundamental changes in response to shifting social, technological, economic, environmental, and political contexts. These shifts have led to further developments in the workplace and work, with implications for the safety, health, and well-being of the workforce [1]. As a result, experts have called for innovative and responsive occupational safety and health (OSH) approaches to better understand burgeoning future of work issues, help harness presented opportunities, and address exhibited challenges [2,3,4,5]. One such approach is the *Total Worker Health*^®^ (TWH) framework [6,7], which recognizes work as a social determinant of health and prioritizes the principles of healthy work design [8] to protect and promote worker well-being both on- and off-the-job. Such holistic OSH strategies can improve our capacity to confront anticipated and unanticipated hazards and exposures likely to meet tomorrow’s workforce, thereby advancing worker well-being, overall. Consequently, workers and employers will be more likely to thrive and productively contribute to their communities and the Nation.

## 2. Objective of the Special Issue

The collection of articles, reviews, and commentaries from the USA presented in this special issue [9] add to the worker safety, health, and well-being literature by focusing on existing or expected future of workplace and work factors, their implications for the future workforce, and expanded OSH strategies and methods to address them.

## 3. The Papers

A total of 29 papers that underwent the IJERPH peer review process were accepted for publication in this special issue, based on scientific quality and methodological soundness. These papers cover a far-reaching range of workplace settings; work functions; worker populations; OSH risks, hazards, and exposures; safety, health, and well-being issues and outcomes; research methods, strategies, interventions, and practical applications; and tools, metrics, guidelines, and other resources. In this editorial, the guest editors offer an introduction to each, organized by type (research, review, and other), as ordered on the corresponding IJERPH special issue webpage [9].

### 3.1. Research

Continuing to build the evolving future of work evidence base and its OSH strategies is essential. Featured research papers focus specifically on either the TWH approach or the development or application of healthy work design strategies to address a variety of future of work topics, offering insights to OSH and related professionals and practitioners to help inform their own research and practice efforts. In this section, papers are organized into five interrelated sub-sections: social context and factors, leadership, small businesses, organizational design, and mixed methods and qualitative methods approaches.

#### 3.1.1. Social Context and Factors

The social context—networks and support derived from a variety of sources including through work—and social factors that may increase vulnerability are critical well-being contributors, providing an avenue for potential OSH opportunities, especially important for higher risk sectors and during times of crises.

To begin, Namazi et al. [10] and Kotejoshyer et al. [11] separately discuss aspects of the development, implementation, and evaluation of a TWH peer health mentoring program designed to address safety, health, and well-being for newly hired correctional officers (CO), a worker population at increased risk of issues such as cumulative stress and resultant outcomes. Though overall CO physical and mental health decline during the study, participating mentors note several social benefits of the program, such as opportunities to learn more about themselves and form meaningful relationships with co-worker mentees.

Horan et al. [12] also offer a peer mental health support intervention, in this instance for first responders, a worker population that suffers from a high burden of chronic stress and mental health distress from critical incidents. While findings suggest a floor effect for self-efficacy, the authors conclude that trust, attention to the social context, and other collaborative safety and health solutions will be vital in light of future of work changes.

Haas et al. [13] advocate for more strategic planning after examining emergency response calls from fire departments during the COVID-19 pandemic. The authors find higher social vulnerability based on household composition, minority/language, and housing/transportation increase the risk of first responders’ exposure to SARS-CoV-2 (the virus that causes COVID-19), and are significantly associated with response calls that require emergency treatment and transport.

#### 3.1.2. Leadership

Employers, and upper and middle management play a pivotal role in the lives of workers. Healthy leadership is therefore essential not only for the well-being of the workforce but also for that of organizations.

Cavallari et al. [14] pilot test HearWell, a TWH intervention created to preserve hearing among highway maintainers. The participatory research approach identifies a need for a comprehensive safety leadership training for managers to be included within existing program elements, emphasizing the importance of healthy leadership within hearing conservation interventions.

Relatedly, Rohlman et al. [15] evaluate a TWH on-line training for supervisors of young agricultural workers, considered at greater risk of injury compared to young workers in other industries. Post-training, the authors observe that supervisors have a greater understanding of the risks to young workers and are more likely to engage in communication behaviors to protect their safety and health.

Collins and colleagues [16] survey directors of nursing at nursing homes, using the Workplace Integrated Safety and Health (WISH) instrument to assess TWH polices, programs, and practices related to leadership commitment, participation, supportive working conditions, comprehensive and collaborative strategies, and adherence to federal and state regulations and ethical norms. Findings reveal high implementation of TWH approaches, though the authors are unable to distinguish certain types of nursing homes that might benefit from additional TWH efforts.

Nagler et. al. [17] use the TWH Implementation Guidelines to evaluate the TWH approach for low-wage food service workers. Site-level managers, different levels of leadership, and frontline workers from a large multinational company are involved in the development and implementation of this intervention designed to address high levels of injury and turnover. Results support the TWH Implementation Guidelines as a useful and likely transferable resource for other industries and similar interventions.

#### 3.1.3. Small Businesses

Unlike many larger organizations, small businesses are often faced with limited resources, making it more challenging to comprehensively meet changing workforce needs, and reinforcing evidence that a one size approach does not fit all.

Macy et al. [18] focus on the state of Kentucky to examine trends in organizations offering workplace health promotion and OSH programs and identify potential program-related gaps and disparities. Though more employers report making such programs available compared to previous years, larger workplaces remain more likely to offer them as opposed to smaller ones.

Similarly, Cunningham et al. [19] apply the NIOSH Small Business Intervention Diffusion Model to conduct community-based activities in two metropolitan communities, assessing perceptions of a TWH approach in small businesses. The authors observe that intermediary organizations such as local health departments and workers compensation insurers find value in providing TWH assistance to small employers, but several safety and health intervention challenges remain, especially inadequate resources.

Brown and colleagues [20] examine whether employees in small businesses receiving a TWH leadership development intervention prior to the COVID-19 pandemic are able to maintain pre-COVID-19 pandemic perceptions of safety and health climates and well-being. Though employees view their workplaces as supportive of their safety and health, other factors continue contributing to a decline in worker well-being during the COVID-19 pandemic.

#### 3.1.4. Organizational Design

The overall context in which work is performed determines the hours, shifts, load, control, flexibility, and arrangements that significantly contribute to worker safety, health, and well-being outcomes. These facets of organizational design have not only been increasingly spotlighted in the future of work but have also experienced accelerated changes since the COVID-19 pandemic.

Ray and Pana-Cryan [21] analyze data from the 2002–2018 General Social Survey—Quality of Worklife and conclude that working at home increases the likelihood of job stress and job satisfaction; taking time off decreases the likelihood of job stress and days with activity limitations, and increases the likelihood of job satisfaction; and adopting a more flexible schedule decreases the likelihood of job stress and increases the likelihood of job satisfaction.

Hernandez et al. [22] examine how frequencies of high workload and recovery activities from work and non-work are associated with same day well-being measures. The authors find that more frequent engagement in high workload activities is associated with lower well-being (i.e., higher stress), while greater recovery activity frequency is associated with higher well-being (i.e., lower stress and higher positive affect).

Davis and Rohlman [23] investigate motor vehicle crash data and county-level characteristics and determine that winter weather that produces hazardous driving conditions also plays a role in crashes during commute hours. The authors suggest adopting policies for flexible work start and stop times, or remote work, to help workers avoid associated risks.

And, Tumlin et al. [24] investigate equine-assisted programs that address human health issues and rely on a predominantly volunteer workforce (not often represented in OSH) with non-traditional schedules, to assess protections for worker well-being in environments characterized in part by dust exposure. The authors identify factors needed to refine the scalability of future air contaminant monitoring and provide frameworks for education, workplace design, and prevention of exposure to dust.

#### 3.1.5. Mixed Methods and Qualitative Methods Approaches

While most studies in this special issue take a quantitative methods approach, mixed methods and qualitative methods are particularly useful in elucidating the complex and novel concerns workers face that can help in the development of innovative OSH strategies.

For example, Hebert-Beirne et al. [25] use a mixed methods community health assessment in high social and economic hardship neighborhoods with a high proportion of individuals engaged in precarious work. Based on themes extracted from focus group data, the authors advocate for resident worker voices to be heard and the imperativeness of their participation in all efforts that impact them to better ascertain nuanced issues and establish a culture of healthy work.

Hudson et al. [26] explore how the Hierarchy of Controls Applied to NIOSH Total Worker Health (TWH HoC) are implemented, focusing specifically on work-related issues of fatigue, stress, sedentary work, and tobacco control. Key determinants identified include organizational culture, implementation climate, and readiness for implementation. The authors emphasize the need to better understand the extent to which OSH guidance like the TWH HoC is used to help prepare for future opportunities and challenges.

Fukumura and colleagues [27] collect worker perspectives on incorporating artificial intelligence (AI) in office settings using a qualitative study design. Based on their findings, the authors note that acceptability of AI in the workplace is complex, that divergent worker-level needs must be addressed, and that AI acceptance is dependent upon whether advantages outweigh challenges. These will remain paramount considerations as new and emerging technologies are more widely applied across workplaces, particularly as employers consider adopting hybrid or remote flexible work options.

Pratap et al. [28] perform an environmental scan of unemployment and underemployment via a literature review and key informant interviews. The authors highlight the pivotal role that employment (and lack thereof) plays in the work and home lives of individuals and the need to prioritize integrated workforce health and well-being approaches into labor and economic development agendas to reduce health inequities.

Peters et al. [29] share outcomes from a literature review, workshop, interviews with experts, and cognitive testing conducted to develop a measure that assesses “thriving” at work. The measure is defined as “the state of positive mental, physical, and social functioning in which workers’ experiences of their work and working conditions enable them to thrive in their overall lives, contributing to their ability to achieve their full potential in their work, home, and community”.

An additional article by Peters and other colleagues [30] uses focus groups and key informant interviews as part of a larger study that develops and evaluates a TWH intervention to examine work’s role in fatigue, stress, back pain, and poor dietary habits among professional bus drivers. Primary themes of the study include lack of trust between drivers and supervisors, scheduling of shifts and routes, and difficulty performing positive health behaviors.

Using a case study as an example, Tenney et al. [31] discuss the role of dissemination and implementation (D&I) science as a critical piece in the translation of research into practice to flexibly implement core TWH intervention components. The authors highlight one D&I framework in particular: Reach, Effectiveness, Adoption, Implementation, and Maintenance (RE-AIM), useful across TWH implementation phases and for employers with varying contextual factors, such as business size and industry type.

### 3.2. Reviews

Featured review papers focus on several key future of work topical areas, offering readers comprehensive background and guidance to enhance the role of OSH as the world of work unfolds.

To help inform policymakers and employers, Ng et al. [32] examine pre-COVID-19 pandemic and COVID-19 pandemic trends for three topics (work arrangements, compensation and benefits, and the organization of work) to explore their effects on the future of work. The authors find that the COVID-19 pandemic has both accelerated and disrupted various trends, including exacerbating work-related inequities.

Pishgar and colleagues [33] use the Risk Evolution, Detection, Evaluation, and Control of Accidents (REDECA) framework across five occupational sectors to review the literature and evaluate AI’s use in detecting hazards, incidents, and harmful work conditions. The authors call for additional exploration of AI’s expanded use to enhance worker well-being.

Horan and another set of co-authors [34] contend that sound recommendations for the future of work need to account for the overall context of the specific industry in question. Using the hospitality industry as an example, the authors describe the advantages of the industry context perspective; examine present trends and the extent to which the OSH literature incorporates an industry focus; and provide informed solutions for researchers and practitioners to better understand and address developing OSH issues.

Given the unknowns brought on by workplace, work, and workforce developments and the increasing implementation of TWH approaches, Rogers and Schill [35] review TWH ethical constructs and competencies and examine codes of ethics for workers in OSH, health promotion, and education. Using an ethical decision-making case study, the authors analyze crucial issues for TWH and propose a transdisciplinary framework that supports both elements of an ethical workplace culture and a core set of practical ethical competencies.

Streit et al. [36] demonstrate the need to employ innovative methods and approaches to monitor and prepare for the emergence of safety and health issues and trends. The authors review strategic foresight—a promising methodological tool that can enhance OSH capacity to anticipate, and perhaps even shape, the future of work—and present a working foresight framework tailored for OSH, with recommendations for research and practice.

### 3.3. Other

In this third and final section of the special issue, featured “other” papers consist of commentaries that provide poignant considerations when exploring current and emerging future of work issues and the expanded approaches used to address them.

Guerin et al. [37] examine D&I science for TWH researchers and practitioners, offering an overview of the literature; a plain language explanation of key concepts; a TWH research-to-practice case study example; and a discussion of future opportunities to increase worker safety, health, and well-being through D&I science.

Flynn et al. [38] discuss existing and persistent OSH inequities, calling for more holistic OSH approaches that account for the social contexts within which work-related injuries and illnesses occur, especially given COVID-19 pandemic impacts. The authors cover several areas to advance health equity, including a biopsychosocial paradigm tool that explores the dynamic, multidirectional interactions between biological phenomena, psychological factors, and social contexts.

## 4. Conclusions

A culture of safer and healthier work will require preparation, vigilance, and a willingness to continuously adapt. As the future of work evolves, so will complex factors that influence worker safety, health, and well-being outcomes and needed OSH research, strategies, interventions, methods, and tools. The papers featured in this special issue offer readers an opportunity to explore a range of issues that currently exist and ones that may emerge, as well as healthy work design and well-being strategies that can be used to address them. These may not only inspire new research on the future of work, but also inform practice, policy, and capacity-building activities. Next step efforts will especially benefit from healthy leadership, active worker participation, customized and responsive interventions, and proactive and collaborative partnerships. Ideally, employers, workers, governments, academic institutions, industry, not-for-profit businesses, labor unions, community organizations, and international establishments will pull together to successfully navigate the future of work and the future practice of OSH.
